# Predictive Role of Biopsy Based Biomarkers for Radiotherapy Treatment in Rectal Cancer

**DOI:** 10.3390/jpm10040168

**Published:** 2020-10-13

**Authors:** Yugang Wen, Senlin Zhao, Annica Holmqvist, Victoria Hahn-Stromberg, Gunnar Adell, Birgitta Holmlund, Surajit Pathak, Zhihai Peng, Xiao-Feng Sun

**Affiliations:** 1 Department of General Surgery, Shanghai General Hospital, School of Medicine, Shanghai Jiaotong University, Shanghai 200080, China; wenyg1502@hotmail.com (Y.W.); sunshinezsl1989@163.com (S.Z.); 2 Department of Oncology and Department of Biomedical and Clinical Sciences, Linköping University, SE-581 83 Linköping, Sweden; Annika.Holmqvist@regionostergotland.se (A.H.); gunnar.adell@regionostergotland.se (G.A.); Birgitta.Holmlund@regionostergotland.se (B.H.); 3 Department of Medical Cell Biology, Uppsala University, SE-751 05 Uppsala, Sweden; victoriastromberg@hotmail.com; 4 Faculty of Allied Health Sciences, Chettinad Academy of Research and Education, Kelambakkam, Chennai 603 103, India; surajit.pathak@gmail.com

**Keywords:** biomarker, biopsy, radiotherapy, rectal cancer, prognosis

## Abstract

Background and Purpose: Radiation therapy has long been contemplated as an important mode in the treatment of rectal cancer. However, there are few ideal tools available for clinicians to make a radiotherapy decision at the time of diagnosis for rectal cancer. The purpose of this study was to assess whether biomarkers expressed in the biopsy could help to choose the suitable therapy and provide predictive and/or prognostic information. Experimental Design: In total, 30 biomarkers were analyzed in 219 biopsy samples before treatment to discover the possibility of using them as an indicator for radiotherapy selection, diagnosis, survival and recurrence. Results: Twenty-two biomarkers (COX2-RT, COX2-NonRT, etc.; 36.67%) had diagnostic value. For survival, four biomarkers (NFKBP65, p130, PINCH and PPAR) were significant in regulating gene promoter activity and overall survival, while four had a trend (AEG1, LOX, SATB1 and SIRT6). Three biomarkers (COX2, PINCH and WRAP53) correlated with disease-free survival, while eight had a trend (AEG1, COX2, Ki67, LOX, NFKBP65, PPAR and SATB1). Four biomarkers (COX2-RT, NFKBP65cyto-RT, P130cyto-NonRT and PPARcyto-RT) were independent prognostic factors for recurrence. NFKBP65 and SIRT6 were significantly correlated with lymph node metastasis regardless of radiation. Patients with high AEG1, LOX, NFKBP65, PPAR and SATB1 had or showed a positive trend for better survival after radiotherapy, while those with positive PINCH and WRAP53 expression would not benefit from radiotherapy. Conclusions: AEG1, LOX, NFKBP65cyto, PPAR and SATB1 could be used as indicators for choosing radiotherapy. COX2-RT, COX2-NonRT and some other biomarkers may provide additional help for diagnosis.

## 1. Translational Relevance

Multidisciplinary management has improved the prognosis of rectal cancer in recent years. The role of radiotherapy in the treatment of rectal cancer has also been initiated in the last few decades. Advances in preoperative radiotherapy have reduced the disease relapse and markedly increased the survival. However, there are few ideal tools available for the clinicians to make a treatment decision at the time of diagnosis. In this study, we analyzed the Astrocyte elevated gene-1 (AEG-1), CD163, COX2, Forkhead box M1 (FOXM1), ki-67, LIVIN, lysyl oxidase (LOX), Musashi-1 (MSI-1), nuclear factor-kappa Bp65 (NFKBP65), particularly interesting new cysteine-histidine-rich protein (PINCH), peroxisome proliferator-activated receptor (PPAR), PRb2/P130, p53, p73, phosphatase of regenerating liver (PRL), RNA-binding motif protein 3 (RBM3), special AT-rich sequence binding protein 1 (SATB1), sirtuin 6 (SIRT6), Tafazzin (TAZ) and WRAP53 biomarkers in 219 biopsy samples before treatment to discover the possibility of using them as an indicator for radiotherapy selection, diagnosis and prognosis. The results indicate that five biomarkers could serve as potent indicators for radiotherapy. Some biomarkers may provide additional aid for diagnosis, especially for those cases that are difficult to draw conclusions on with the traditional Hematoxylin–Eosin staining. Only a few biomarkers could provide prognostic information for survival or recurrence, suggesting that it is unsuitable to predict prognosis excessively depending on the biomarkers in the biopsy. This study provides valuable information for the predictive or prognostic significance of biomarkers in biopsy and may give useful information to clinical practice.

## 2. Introduction

Colorectal cancer is among the three most common cancer diagnoses and causes of cancer death in women and men in Sweden and China [1,2]. Multidisciplinary management has been implicated to improve the prognosis in recent years [3,4]. Preoperative radiotherapy (RT) is considered to downstage the rectal cancer (RC) and is reportedly helpful to improve the surgical success rates and improve the survival in RC patients [5,6].

Accurate diagnosis and preoperative staging are essential to determine the optimal therapeutic plan for a patient. Computer tomography (CT) or magnetic resonance imaging (MRI) for polyp characterization is standard for preoperative staging but sub-optimal sensitivity and specificity values for detection of nodal metastasis are reported [7,8,9]. To date, there are few ideal markers available for the clinician to choose the optimal treatment (e.g., neoadjuvant RT or not) at the time of diagnosis for RC. Furthermore, the prognosis of RC patients varies significantly, even for those with the same TNM stage [10]. Therefore, biomarkers for better risk evaluation might be essential to assess the tumor status, identify patients with a high risk of progression and help clinicians make the best therapeutic plan at the time of diagnosis in RC.

Although alterations in numerous genes and pathways such as the APC/β-catenin/Tcf pathway have been indicated as possible risk factors for RC progression, the studies were mainly focused on tumor specimens obtained after operation and might be of little value to the initial management of the patients [11,12]. Biopsy samples before treatment might be more suitable for the assessment of tumor characteristics. Morton et al. [13] discovered in their clinical trial that biopsy-based staging of primary melanomas provided important prognostic information and identified patients with nodal metastases who might benefit from the complete lymph adenectomy; biopsy-based management prolonged disease-free survival (DFS) for all patients and extended distant DFS and melanoma-specific survival for patients with nodal metastases from intermediate-thickness melanomas. Researchers also found that neither the disease course nor the survival rate was affected by the previous biopsy and it could therefore be implemented safely [14]. In primary CRC, the preoperative biopsy is essential for confirmation of diagnosis but currently fails to provide predictive information to clinicians [15].

Assessing the status of biomarkers in biopsy samples may provide additional diagnostic and predictive information, compared to traditional diagnosis with histopathology [16,17]. However, the study on biomarkers in the biopsy of RC is rare and/or with a small sample size [18]. In our previous studies, we detected the expression of 30 biomarkers in biopsy samples (BS) before treatment of RC patients, included in a Swedish clinical trial of preoperative RT or a Dutch rectal cancer trial [19,20,21,22,23]. In the present study, we analyzed all available data in Linköping University Hospital to accurately clarify the predictive value of the biomarkers expressed in the biopsy for diagnosis and prognosis and to access their possibility as RT indicators for RC patients.

## 3. Materials and Methods

### 3.1. Patients and Samples

The study included 219 rectal cancer patients from the South-East Swedish Health Care region who participated in a Swedish clinical trial of preoperative RT or a Dutch rectal cancer trial, as described previously [19,20,21,22,23]. The experimental protocol was approved by Linkoping University, Sweden. The patients were randomized to undergo preoperative RT and surgery (RT group, *n* = 127) or surgery alone (Non-RT group, *n* = 92). RT was given at 25 Gy in 5 fractions during a median of 6 days (range, 5–12 days). Surgery was performed within a median of 8 days after RT (range, 1–15 days). The expression of different biomarkers in biopsy samples (BS, the sampling before the operation) and in surgical samples including distant normal mucosa (DN, histologically free from tumor cells), primary tumor (PT) and lymph node metastasis (LNM) were determined by immunohistochemistry. The number of the samples varied for different biomarkers depending on available pathological material. The required informed consent was given by all participants. None of the patients received preoperative or adjuvant chemotherapy. There were no significant differences between the RT group and Non-RT group concerning the clinicopathological characteristics (Supplementary Table S1). All methods were performed per the relevant guidelines and regulations of Linkoping University, Sweden.

### 3.2. Immunohistochemistry

Immunohistochemistry (IHC) was performed at our laboratory for the following proteins: astrocyte elevated gene-1 (AEG-1, also called MTDH, primary rabbit polyclonal anti-MTDH antibody, Zymed, San Francisco, CA, USA), CD163 (mouse monoclonal antibody, Novocastra, England), COX2 (polyclonal antibody, Cayman Chemical, Ann Arbor, MI, USA), Forkhead box M1 (FOXM1), Ki-67 (mouse monoclonal antibody MIB-1, Immunotech, SA, Marseilles, France), LIVIN, lysyl oxidase (LOX, polyclonal rabbit antibody, synthesized by Open Biosystems), Musashi-1 (MSI-1, rabbit monoclonal antibody, EMD Millipore, Billerica, MA, USA), nuclear factor-kappaBp65 (NFKBP65, rabbit anti-phospho-Ser536-p65 antibody, Abcam, MA, USA), particularly interesting new cysteine-histidine-rich protein (PINCH, rabbit polyclonal antibody, a gift from Prof. Ann Rearden, Department of Pathology, University of California, CA), peroxisome proliferator-activated receptor (PPAR, polyclonal rabbit, ARP37889, against N terminal; Aviva Systems Biology, San Diego, CA, USA), PRb2/P130 (mouse monoclonal antibody, Neomarkers, Portsmouth, NH, USA), P53 (rabbit polyclonal anybody, Novocastra Laboratories Ltd., Newcastle upon Tyne, UK), P73 (goat polyclonal antibody, Dako, Glostrup, Denmark), phosphatase of regenerating liver (PRL, rabbit polyclonal antibody, a giftfrom Professor Bert Vogelstein, Johns Hopkins University Medical Institutions, Baltimore, MD, USA), RNA-binding motif protein 3 (RBM3, mouse monoclonal antibody, Atlas Antibodies AB, Stockholm, Sweden), special AT-rich sequence binding protein 1 (SATB1, Rabbit Monoclonal Antibody, Abcam, Cambridge, MA, USA), sirtuin 6 (SIRT6), Tafazzin/wwtr1 (TAZ, rabbit polyclonal antibody, HPA039557, Sigma-Aldrich Co, St. Louis, MO, USA) and WRAP53 (rabbitα-WRAP53-C1 polyclonal antibody). The IHC with the different antibodies was performed on the same patient samples at our laboratory. The intensity of staining was classified as 0 (negative staining: ≤5% positive cells), 1 (weak staining: weak yellow), 2 (moderate staining: yellow-brown) and 3 (strong staining: brown) and/or the proportion of staining was scored into five grades as 0%, 1–25%, 26–50%, 51–75% and 56–100%. A case was evaluated as positive if it contained any positive cancer cells and negative otherwise. The staining patterns were graded as cytoplasmic, nuclear or stromal staining. The immunostaining was independently evaluated by two researchers including one pathologist without clinical and pathological knowledge about the patients. In the case of discrepant scoring results, a consensus score was reached after re-examination. The re-analysis was performed as described previously [17,18,19,20,21].

### 3.3. Evaluation of RT Impact on Biomarkers and Potential Value of Biomarkers in BS in Different Groups

We mainly implemented the study on three different levels. Firstly, we evaluated whether the radiation had an impact on the expression of the biomarkers. Secondly, we compared the staining of the different biomarkers BS, DN, PT and LNM between the Non-RT group and RT group, respectively, to see whether there was a correlation among those samples. Thirdly, we compared the expression of biomarkers in matched samples (the samples from the same patients) in Non-RT group (*n* = 63) and RT group (*n* = 56), respectively, to exactly evaluate their predictive and diagnostic significance and, more importantly, find out whether the biomarkers in BS could be used as RT indicators. For the protein expressed in the cytoplasm, nucleus and/or stroma, we distinguishingly named them as “-cyto”, “-nucl” and “-strom” in “-RT” or “-NonRT” group, respectively, e.g., “FOXM1cyto-NonRT” and “FOXM1nucl-RT”. Thus, with 30 biomarkers in each group, in total 60 biomarkers were analyzed.

### 3.4. Statistical Analysis

The clinicopathological characteristics between RT and Non-RT groups were analyzed by Chi-square (χ^2^) test or Fisher’s exact test. For ranked data, a nonparametric test is a suitable statistical option [24] The differences of each biomarker among BS, DN, PT and LNM, as well as their association, were analyzed by nonparametric test—independent samples—Kruskal–Wallis, one-way ANOVA (K samples), Mann–Whitney U test (2 samples) and Spearman bi-variate correlation analysis. For survival analysis, Kaplan–Meier and Cox regression were used. The test was two-sided and *p*-value < 0.05 was considered to be statistically significant. All statistical analyses were performed using IBM SPSS Statistics 23.0 software.

## 4. Results

### 4.1. RT Impact on Biomarkers in PT

**Figure 1:**
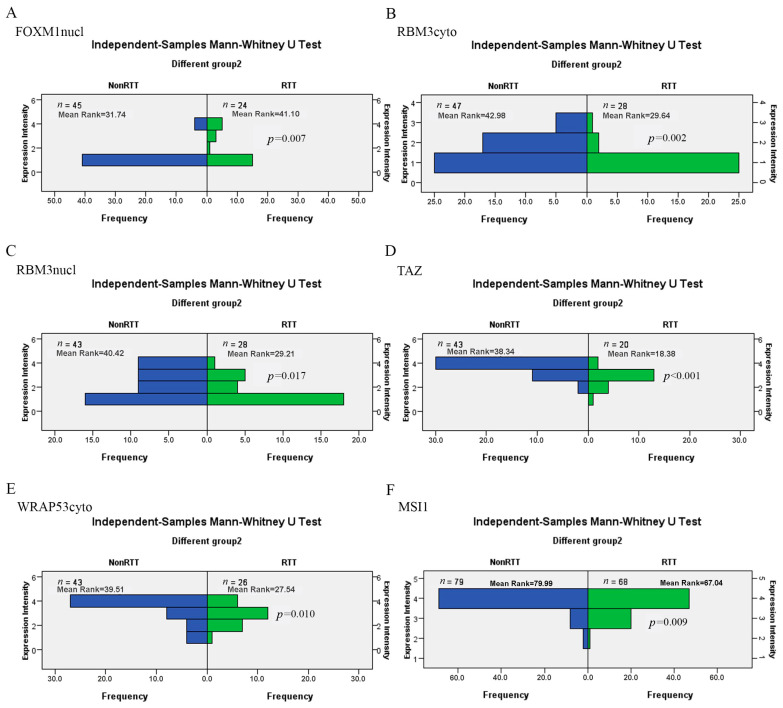
The expression of six markers in primary tumor tissue was affected by RT (*p* < 0.05). (**A**) FOXM1nucl (increased, *p =* 0.007); (**B**) RBM3cyto (reduced, *p =* 0.002); (**C**) RBM3nucl (reduced, *p =* 0.017); (**D**) TAZ (reduced, *p <* 0.001); (**E**) WRAP53cyto (reduced, *p =* 0.010); and (**F**) MSI1 (reduced, *p =* 0.009). Non-RTT, primary tumor in Non-RT group; RTT, primary tumor in RT group; N, number of cases.

The analysis showed that six biomarkers in PT were significantly changed by RT: FOXM1nucl (increased, *p =* 0.007), MSI1 (reduced, *p =* 0.009), RBM3cyto (reduced, *p =* 0.002), RBM3nucl (reduced, *p =* 0.017), TAZ (reduced, *p <* 0.001) and WRAP53cyto (reduced, *p =* 0.010) (Figure 1). The expression of the 24 other biomarkers, compared with those in Non-RT group, was not significantly affected by RT (24/30, 80.00%). The results are consistent with those analyzed by Pearson chi-square (Supplementary Figure S1).

### 4.2. The Differences between BS and PT in the Unmatched or Matched Cases (RT Group or Non-RT Group, Respectively)

We analyzed the difference of 60 biomarkers (i.e., 30 biomarkers each in Non-RT and RT group,) between BS and PT in the unmatched or matched cases to find out whether BS can represent PT, especially focused on those in the matched samples, as the results from the matched samples would be more dependable.

Analyzing biomarkers in BS and PT samples, we found the expression of FOXM1cyto-NonRT, FOXO3Acyto-NonRT, LIVIN-NonRT, MSI1-NonRT, MSI1-RT, NFKBP65cyto-NonRT, NFKBP65cyto-RT, P73cyto-RT, P73cyto-NonRT and TAZ-NonRT in BS were less than those in PT (*p <* 0.05; 10/60, 16.67%), while the expression of the other biomarkers in BS were equal to or more than those in PT tissue (50/60, 83.33%; Supplementary Table S2).

Then, we used correlation analysis to further discover the relationship between BS and PT in the RT group or Non-RT group, respectively. The results show that most biomarkers in BS were related to PT, of which 24 biomarkers had close relationships (Spearman correlation index > 0.3, *p <* 0.05; data not shown). This additionally confirmed that, in most cases, biomarkers in BS could be those of PT.

### 4.3. Diagnostic Value of Biomarkers in BS

We compared the expression of biomarkers in BS, DN, PT and LNM samples overall in Non-RT or RT group, respectively, and found that 13 of them had no difference (13/60, 21.67%). In other words, they were not suitable for diagnostic analysis since there was no noticeable difference between BS and DN. The other 47 biomarkers were different when compared (47/60, 78.33%) and would be further analyzed for their diagnostic value. Comparing the expression of biomarkers between BS and DN highlighted that 22 biomarkers (COX2-RT, COX2-NonRT, etc.) in BS had diagnostic value for RC patients preoperatively (the expression of which in BS was higher than that in DN, *p <* 0.05; 22/60, 36.67%; Table 1). For four biomarkers, CD163-NonRT, LIVIN-RT, RBM3nucl-RT and SATB1-NonRT, *p*-value was in the range of 0.05–0.1, thus these biomarkers probably have the diagnostic significance, and more samples might be needed for further verification. The remaining 21 biomarkers in BS were unsuitable for diagnosis. Furthermore, among those biomarkers affected by RT, MSI1 had diagnostic significance in both Non-RT and RT groups, TAZ only had diagnostic value for Non-RT patients and RBM3nucl had a diagnostic tendency in the RT group.

### 4.4. The Prognostic and Predictive Value of Biomarkers in BS

On comparing the expression of biomarkers between BS and LNM, we found 12 biomarkers in BS were less than those in LNM (*p <* 0.05), while the other 48 biomarkers in BS were equal to or higher than those in LNM (80.00%). Further correlation analysis highlighted that, among the 48 biomarkers, six were closely related to those in LNM (Supplementary Table S3). The results indicate that, regardless of RT, the expression of NFKBP65nucl, SIRT6cyto and SIRT6nucl in BS significantly correlated with those in LNM.

**Figure 2:**
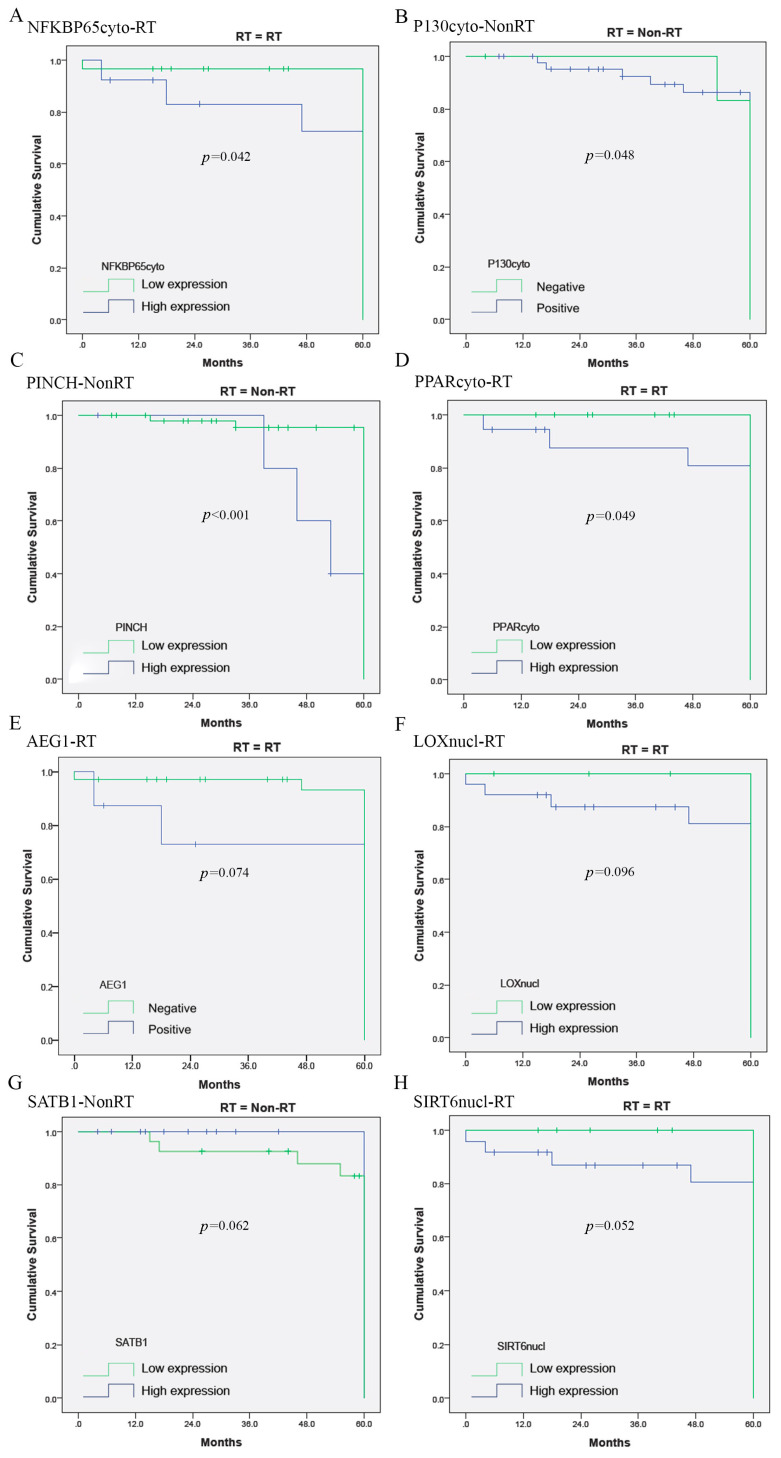
Survival analysis showed that four biomarkers in BS were significantly related to overall survival (**A**–**D**), while AEG1-RT ((**E**), *p* = 0.074), LOXnucl-RT ((**F**), *p* = 0.096), SATB1-NonRT ((**G**), *p* = 0.062) and SIRT6nucl-RT ((**H**), *p* = 0.052) tended to be related to overall survival.

Survival analysis showed that four biomarkers in BS significantly correlated with OS: NFKBP65cyto-RT (*p =* 0.042), P130cyto-NonRT (*p =* 0.048), PINCH-NonRT (*p <* 0.001) and PPARcyto-RT (*p =* 0.049). AEG1-RT (*p =* 0.074), LOXnucl-RT (*p =* 0.096), SATB1-NonRT (*p =* 0.062) and SIRT6nucl-RT (*p =* 0.052) tended to be related to OS. The other biomarkers in BS had no prognostic value for OS (52/60, 86.67%; Figure 2 and Table 2).

Among the 60 biomarkers, the following three were related to DFS: COX2-RT (*p =* 0.022), PINCH-NonRT (*p =* 0.005) and WRAP53cyto-NonRT (*p =* 0.007). AEG1-RT (p *=* 0.091), COX2-NonRT (*p =* 0.084), Ki67-NonRT (*p* = 0.089), LOXnucl-RT (*p =* 0.088), NFKBP65cyto-RT (*p =* 0.054), PPARcyto-RT (*p =* 0.067), SATB1-NonRT (*p =* 0.094) and WRAP53nucl-NonRT (*p =* 0.077) had the tendency to be related to DFS. Patients with positive expression of these biomarkers had reduced DFS time compared to those with negative tumors (Supplementary Figure S2 and Table 3). The other 49 biomarkers in BS had no prognostic value for DFS (49/60, 81.67%).

Multivariate Cox proportional hazard models analysis indicated that COX2-RT (HR: 2.747, 95% CI: 1.733–3.562, *p <* 0.001), NFKBP65cyto-RT (HR: 1.922, 95% CI: 1.507-3.338, *p <* 0.001), P130cyto-NonRT (HR: 1.897, 95% CI: 1.679–2.516, *p <* 0.001) and PPARcyto-RT (HR: 2.910, 95% CI: 2.091–4.830, *p <* 0.001) in BS were independent prognostic factors for local/distal recurrence; patients with high expression of these four biomarkers in BS were more prone to suffer from tumor recurrence (Supplementary Table S4). Eight biomarkers were related to local/distal recurrence, while the remaining 48 biomarkers had no relationship with recurrence (48/60, 80.00%).

**Figure 3:**
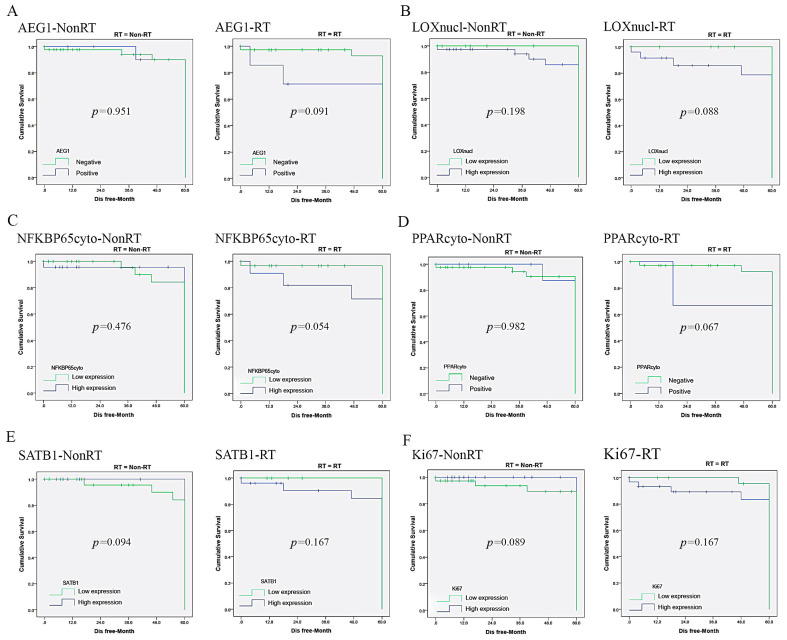
The patients with the high expression of: AEG1 (**A**); LOXnucl (**B**); NFKBP65cyto (**C**); PPAR cyto (**D**); SATB1 (**E**); and Ki67 (**F**), in BS tended to benefit from RT for DFS.

By comprehensive analysis, we discovered that the patients with high COX2 or WRAP53cyto expression in BS tended to have worse OS and DFS, with or without radiotherapy, while high P130cyto expression trended to predict better OS and DFS in Non-RT and RT group. The patients with positive AEG1, LOXnucl, NFKBP65cyto, PPARcyto and SATB1 had or trended to have better OS and DFS after RT (Figure 3 and Supplementary Figure S3), positive Ki67 expression trended to have close correlation with better DFS after RT and those with positive SIRT6nucl trended to have a better OS after RT. Conversely, those with high PINCH and WRAP53nucl in BS did not benefit from RT (Supplementary Figure S4); thus, these biomarkers might help to distinguish those who would benefit from RT.

## 5. Discussion

Biopsies, owing to their precise evaluation, are increasingly being used for treatment options and prognostic evaluation in patients with CRC [25] and other tumors [26]. For accurate treatment, tumor biopsy aids as a confirmation for CRC diagnosis. An accurate prognosis is essential for choosing the optimal therapeutic strategy for primary RC. According to the results of the Swedish clinical trial, preoperative RT compared to chemotherapy can reduce the risk of local recurrence and improve survival after curative surgery for RC [8,27]. Reliable biomarkers based on pretreated biopsy specimens of RC are currently missing, especially for prognosis. The provocation for the clinician and pathologist will be to decide and validate recent prognostic and predictive biomarkers by utilizing pretherapeutic biopsies. Can the biomarkers expressed in BS be used as an indicator for choosing a suitable therapy regimen or only to provide diagnostic and/or prognostic help? In the present study, we examined 30 biomarkers in pretreated biopsy samples, trying to clarify this question. To the best of our knowledge, this is the first report analyzing so many biomarkers in BS of RC patients. We came across several biomarkers that could be used as RT indicators.

Initially, we evaluated the impaction of RT on the expression of the 30 biomarkers in PT. The results show that the expressions of most biomarkers (80.00%) were not considerably changed by RT, while six biomarkers were significantly increased or reduced in PT after preoperative RT: FOXM1nucl, MSI1, RBM3cyto, RBM3nucl, TAZ and WRAP53cyto. Among them, MSI1 had diagnostic significance in both the Non-RT and RT groups; RBM3nucl trended to have diagnostic significance in patients who received RT; TAZ was helpful for the diagnosis in the Non-RT group; and WRAP53cyto was related to DFS and had a trend with recurrence in Non-RT group patients. We then analyzed the varied expression of biomarkers between BS and PT in different levels (RT group, Non-RT group and the matched data) to observe whether BS could represent PT. We observed that the expression of 10 biomarkers in BS was less than those in PT, while the 50 other biomarkers (83.33%) expressed in BS were equal to one or more of those in PT. Correlation analysis showed that most biomarkers in BS were related to PT, of which 24 biomarkers had a close relationship. The results further confirm that, in most cases, BS markers could be of PT, although in 16.67% of cases BS was less than PT.

In the last few years, several studies have demonstrated high concordance between biopsy and resection samples in CRC and other cancers using IHC and molecular testing such as cytogenetic testing and microarray analysis [23]. Shia et al. [28] analyzed the IHC staining patterns for MLH1, MSH2, MSH6 and PMS2 in the matched biopsy and resection samples in 70 carcinomas of the tubular gastrointestinal tract and confirmed that biopsy samples were as reliable as resection samples in predicting mismatch repair protein abnormality. However, Waaijer et al. [29] found that decision of adjuvant treatment based on core needle biopsy (CNB) would have resulted in overtreatment in seven (3.5%) and under-treatment in three (1.5%) of 199 breast cancer patients; the substantial difference in tumor grading between CNB and resection samples affected the indication for adjuvant therapy, although only in a small minority of patients.

The difference might be caused by multiple factors: (1) It could be a result of the less tissue sampled for the biopsy, i.e., the tissue obtained may be insufficient to represent the overall tumor, in addition to CRCs being known for their intra-tumor heterogeneity [30]. The utilization of preoperative diagnostic biopsies would thus seem to be prone to false-negative errors. (2) There could be a failure to detect a representative part of the tumor because of the limitation of the detection method used. Fine needle aspiration (FNA) biopsy, used for lumps characterized as palpable masses, had less diagnostic accuracy than CNB; the use of CNB should be encouraged when the initial FNA is inconclusive or methods should be improved by using 12-core systematic biopsy to get more tissue samples [31]. Tang et al. [32] found the accuracy for CNB was significantly higher than forceps biopsy (76.7% vs. 36.1%; *p <* 0.001) in RC patients. Routine forceps biopsy, causing tissue fragmentation in a few cases, was of limited value especially in identifying residual cancer cells after nCRT. (3) The third caveat relates to the possibility that some of the driver mutations are occasionally selected late, and may thus be presented in a sub-clone that has not yet gained dominance when the biopsy is carried out, although that might happen at a very low frequency. (4) IHC performed in tissue microarrays rather than whole-tissue sections may also, to a small extent account, for the different percentages of positive results [33]. (5) It might be because of the controlled hypo-perfusion during operation to reduce blood loss by the anesthetist and reduced vascularity to avoid the spread of tumor cells by prior ligation of vessels and intestine, which may partly contribute to the difference between biopsy and PT.

Rossum et al. [34] found the pooled estimate for the sensitivity of endoscopic biopsy after nCRT in esophageal cancer was 34.5%. Therefore, complete reliance on negative endoscopic biopsy findings for deciding to postpone surgery would likely result in local recurrences in the majority of patients. Endoscopic sampling error due to the irradiated luminal surface with remaining small tumor foci combined with inflammation, fibrosis and the presence of non-mucosal residual cancer in deeper esophageal wall layers were thought to account for the high false-negative results. To improve the sensitivity of the endoscopic biopsy after nCRT, extensive sampling protocols and deeper bite-on-bite submucosal biopsies have been suggested.

On the other hand, if the expression of an oncoprotein in BS is lower than that in DN, it does not possess any diagnostic value. We analyzed the diagnostic value of biomarkers in BS and confirmed that 22 factors (36.67%) could be used as diagnostic biomarkers. Four biomarkers (6.67%) had the tendency to be meaningful for diagnosis and might need more studies for further validation.

Several research groups have indicated the predictive value of biomarkers in the biopsy of various cancers. Sato et al. [35] demonstrated that prostate cancer patients with negative GCNT1 expression in biopsy were associated with significantly better survival rates. Prostate-specific antigen-free survival compared with GCNT1-positive tumors, as well as the GCNT1 expression, was a significant and independent predictor of recurrence after radical prostatectomy, which could be used in pre-treatment decision making for the patient. Wei et al. [18] examined IMP3 expression in the paired biopsy and resection specimens of CRC. They found that IMP3 expression in biopsy was significantly related to lymph node metastasis and stage and had a higher sensitivity than CT scan for lymph nodal metastases, suggesting that IMP3 expression in biopsy was of higher value in detecting lymph nodal metastases and could be used as a potential biomarker for the stage. Similarly, Feng et al. [36] demonstrated that ZEB1 expressions in endometrial biopsy were significantly associated with subtype, grade, myometrial invasion and lymph node metastases. ZEB1 expression could help physicians to better predict the lymph node metastasis in the patients prior to hysterectomy.

In the present study, we revealed that the expression of NFKBP65nucl, SIRT6cyto and SIRT6nucl in BS significantly correlated with those in LNM regardless of RT. Four biomarkers in BS correlated with OS: NFKBP65cyto-RT, P130cyto-NonRT, PINCH-NonRT and PPARcyto-RT. AEG1-RT, LOXnucl-RT, SATB1-NonRT and SIRT6nucl-RT tended to be related to OS. The other 86.67% (52/60) of biomarkers had no predictive value for OS. Among the 60 biomarkers, three were related to DFS: COX2-RT, PINCH-NonRT and WRAP53cyto-NonRT. AEG1-RT, COX2-NonRT, Ki67-NonRT, LOXnucl-RT, NFKBP65cyto-RT, PPARcyto-RT, SATB1-NonRT and WRAP53nucl-NonRT tended to be related to DFS. The patients with positive expression of these biomarkers were observed to have reduced DFS time compared to those with negative tumors. The other 49 biomarkers (81.67%) had no predictive significance for DFS. Further analysis indicated that only four biomarkers (6.67%), COX2-RT, NFKBP65cyto-RT, P130cyto-NonRT and PPARcyto-RT, in BS were independent prognostic factors for local/distal recurrence; the patients with high expression of these biomarkers were more prone to suffer from tumor recurrence. Eight biomarkers had the trend to be related to local/distal recurrence; however, there was no relationship between the other 48 biomarkers (80.00%) and recurrence. The patients with high COX2 or WRAP53cyto expression tended to have worse OS and DFS regardless of RT, while high P130cyto in BS tended to predict better OS and DFS in both Non-RT and RT groups. Importantly, patients with positive AEG1, LOXnucl, NFKBP65cyto, PPARcyto and SATB1 expression had or tended to have better OS and DFS after RT, positive Ki67 expression tend to have a close correlation with better DFS after RT and those with positive SIRT6nucl expression tend to have a better OS after received RT. In contrast, patients with high PINCH and WRAP53nucl did not benefit from RT; thus, these biomarkers might be used as RT indicators.

The present analysis confirmed that: (1) Well-known oncogenes including AEG1, LOXnucl, NFKBP65cyto, PPARcyto and SATB1 can also be used as indicators for RT selection. (2) Only a few biomarkers in BS could provide predictive information for survival or recurrence, so we should be vigilant of their limited value. It might be unsuitable to predict prognosis egregiously depending on the expression of biomarkers in BS. (3) Some biomarkers could provide additional information and be helpful for diagnosis, especially for those cases on which it is difficult to draw diagnostic conclusions with routine Hematoxylin–Eosin staining. As many biomarkers in BS were not meant for diagnosis, it is inappropriate for the clinicians to excessively rely on biomarkers in BS to make their diagnosis. (4) Biopsy has limitations in clinical practice, which might be unavoidable but could be improved. Clinicians should be aware of this and reasonably use it.

There are several limitations to the present study. The sample size was not large enough due to the fact that biopsy samples were not available for all the subjects we selected from the local cancer registry, which might be not reprehensive of the entire corresponding populations. In addition, due to the paucity of biopsy samples, it was not possible to analyze all the selected biomarkers in all samples. Thirdly, owing to the lack of complete information on the family history, we could hardly evaluate whether there were any special characteristics in hereditary RC such as Lynch syndrome-associated RC.

Our results provide valuable information for refining the previously debatable description of the value of biomarkers in biopsy in RC and would give useful information to clinical practitioners. Further studies, focusing on those screened biomarkers in a larger population, are needed to precisely verify the efficiency of pretreatment biopsy specimens in helping the clinician to make a therapeutic selection and predicting the outcome of RC patients.

## Figures and Tables

**Table 1:** Comparing the expression of biomarkers between biopsy samples and distant normal samples in Non-RT group or RT group to clarify their diagnostic value.

Marker	BS		DN		*p*-Value
	N	Mean Rank	N	Mean Rank	
AEG1-NonRT	58	52.46	46	40.54	0.026
AEG1-RT	42	32.02	37	29.98	0.210
CD163-NonRT	58	53.00	46	49.05	0.080
CD163-RT	43	38.50	36	28.50	0.039
COX2-NonRT	44	30.81	38	42.19	0.001
COX2-RT	41	34.25	35	40.10	0.044
FOXM1cyto-NonRT	56	52.00	46	41.00	0.032
FOXM1nucl-NonRT	54	54.18	46	38.00	<0.001
FOXM1nucl-RT	41	31.24	34	18.50	<0.001
FOXO3Acyto-NonRT	54	56.49	44	32.51	<0.001
XO3Acyto-RT	43	34.05	27	21.72	0.002
Ki67-NonRT	45	55.21	41	52.34	0.346
Ki67-RT	28	32.25	26	30.87	0.259
LIVIN-NonRT	59	57.85	46	35.91	<0.001
LIVIN-RT	42	34.14	30	26.00	0.053
LOXcyto-NonRT	57	45.92	46	47.08	0.811
LOXcyto-RT	40	28.48	22	36.52	0.019
LOXnucl-NonRT	57	44.80	45	47.22	0.613
LOXnucl-RT	42	29.96	23	38.22	0.024
MSI1-NonRT	57	83.55	63	39.64	<0.001
MSI1-RT	43	65.63	55	36.89	<0.001
NFKBP65cyto-NonRT	56	51.54	46	41.46	0.050
NFKBP65cyto-RT	43	35.44	25	35.58	0.975
NFKBP65nucl-NonRT	56	44.43	47	49.51	0.047
NFKBP65nucl-RT	43	34.56	24	35.46	0.675
P73cyto-NonRT	58	28.50	20	37.30	0.012
P73cyto-RT	48	24.32	21	36.10	0.001
P130cyto-NonRT	52	40.95	20	40.05	0.820
P130cyto-RT	42	30.91	21	32.09	0.699
PINCH-NonRT	56	51.29	27	38.71	0.026
PINCH-RT	48	46.25	21	27.55	0.016
PPARcyto-NonRT	54	53.08	43	33.15	<0.001
PPARcyto-RT	42	32.57	27	23.26	0.022
RBM3cyto-NonRT	58	57.81	47	37.98	<0.001
RBM3cyto-RT	41	33.22	28	22.96	0.001
RBM3nucl-RT	42	31.94	28	24.20	0.060
SATB1-NonRT	51	45.50	41	37.50	0.068
SATB1-RT	40	31.85	30	30.12	0.616
SIRT6cyto-NonRT	55	56.95	47	37.27	<0.001
SIRT6cyto-RT	44	39.84	24	29.96	0.008
SIRT6nucl-NonRT	53	47.39	46	45.61	0.737
SIRT6nucl-RT	45	37.19	26	36.81	0.923
TAZ-NonRT	57	55.12	43	31.88	<0.001
TAZ-RT	44	33.18	30	27.82	0.188
WRAP53cyto-NonRT	54	52.87	40	30.31	<0.001
WRAP53cyto-RT	44	31.96	26	21.04	0.006
WRAP53nucl-NonRT	55	47.15	44	40.92	0.231

BS, biopsy sample; DN, distant normal sample; N, number of cases.

**Table 2:** Univariate and multivariate Cox proportional hazard models for overall survival of different biomarkers in biopsies.

	RT Group				Non-RT Group			
	Univariate Analysis		Multivariate Analysis		Univariate Analysis		Multivariate Analysis	
	HR (95% CI)	*p*	HR (95% CI)	*p*	HR (95% CI)	*p*	HR (95% CI)	*p*
AEG1	1.820 (1.213–3.124)	0.045	1.581 (1.108–2.915)	0.074	1.005 (0.521–1.252)	0.681	−	−
CD163	0.960 (0.576–1.345)	0.465	−	−	0.880 (0.462–1.099)	0.442	−	−
COX2	1.233 (0.951–2.218)	0.044	1.088 (0.710–1.632)	0.178	0.861 (0.509–1.417)	0.498	−	−
FOXM1cyto	0.875 (0.665–1.127)	0.546	−	−	0.719 (0.636–1.308)	0.421	−	−
FOXM1nucl	1.265 (0.896−2.127)	0.043	0.768 (0.551–1.301)	0.432	1.287 (0.855–1.973)	0.039	0.815 (0.768–1.545)	0.616
FOXO3Acyto	1.001 (0.601–1.236)	0.687	−	−	1.026 (0.705–1.617)	0.449	−	−
Ki67	0.756 (0.433–1.224)	0.437	−	−	0.712 (0.412–1.156)	0.385	−	−
LIVIN	1.129 (0.711–1.237)	0.700	−	−	0.995 (0.532–1.211)	0.575	−	−
LOXcyto	0.923 (0.494–1.268)	0.543	−	−	0.897 (0.561–1.265)	0.458	−	−
LOXnucl	1.356 (0.987–2.826)	0.037	1.253 (0.914–2.435)	0.096	0.625 (0.419–1.087)	0.444	−	−
MSI1	0.910 (0.523–1.336)	0.249	−	−	0.858 (0.469–1.217)	0.311	−	−
NFKBP65cyto	2.401 (1.832–4.365)	0.011	2.225 (1.787–4.064)	0.042	1.354 (1.017–2.620)	0.027	0.989 (0.659–1.578)	0.590
NFKBP65nucl	0.562 (0.387–0.993)	0.505	−	−	0.611 (0.365–1.120)	0.242	−	−
P53	0.741 (0.411–1.103)	0.314	−	−	0.722 (0.405–1.008)	0.206	−	−
P73cyto	0.891 (0.637–1.124)	0.784	−	−	0.712 (0.440–1.432)	0.483	−	−
P130cyto	1.057 (0.501–1.401)	0.350	−	−	2.656 (1.913–4.016)	0.010	2.532 (1.811–3.989)	0.048
PINCH	0.921 (0.585–1.356)	0.454	−	−	3.541 (1.942–5.308)	<0.001	3.239 (1.901–5.003)	<0.001
PPARcyto	2.587 (1.684–3.889)	0.023	2.565 (1.591–3.776)	0.049	0.543 (0.336–1.028)	0.959	−	−
PPARstrom	0.879 (0.535–1.433)	0.125	−	−	0.885 (0.638–1.584)	0.299	−	−
PRL	0.586 (0.371–1.113)	0.450	−	−	0.595 (0.389–1.215)	0.371	−	−
RBM3cyto	0.506 (0.393–0.904)	0.891	−	−	0.612 (0.387–0.947)	0.832	−	−
RBM3nucl	0.715 (0.421–1.019)	0.966	−	−	0.857 (0.569–1.272)	0.837	−	−
SATB1	0.812 (0.564–1.541)	0.178	−	−	1.350 (1.118–2.469)	0.049	1.251 (0.973–2.061)	0.062
SIRT6cyto	0.602 (0.418–0.902)	0.381	−	−	0.525 (0.381–0.865)	0.451	−	−
SIRT6nucl	1.310 (1.058–2.379)	0.034	1.265 (1.024–1.960)	0.052	0.649 (0.417–1.059)	0.279	−	−
TAZ	0.791 (0.607–1.019)	0.555	−	−	0.541 (0.349–0.945)	0.319	−	−
WRAP53cyto	1.267 (0.826–2.358)	0.042	0.896 (0.356–1.321)	0.662	1.216 (0.959–1.837)	0.048	0.861 (0.510–1.530)	0.510
WRAP53nucl	0.859 (0.538–1.252)	0.678	−	−	0.716 (0.433–1.201)	0.273	−	−
WRAP53stromcyto	0.708 (0.423–1.071)	0.312	−	−	0.641 (0.356–0.952)	0.361	−	−
WRAP53stromnucl	0.856 (0.579–1.134)	0.872	−	−	0.764 (0.496–1.082)	0.160	−	−

CI, confidence interval; HR, hazard ratio.

**Table 3:** Univariate and multivariate Cox proportional hazard models for disease free survival of different biomarkers.

	RT Group				Non-RT Group			
	Univariate Analysis		Multivariate Analysis		Univariate Analysis		Multivariate Analysis	
	HR (95% CI)	*p*	HR (95% CI)	*p*	HR (95% CI)	*p*	HR (95% CI)	*p*
AEG1	1.520 (1.217–2.648)	0.041	1.418 (1.103–2.306)	0.091	0.738 (0.623–1.237)	0.951	−	−
CD163	0.718 (0.465–1.021)	0.320	−	−	0.695 (0.433–1.125)	0.608	−	−
COX2	3.467 (1.725–4.763)	0.008	3.345 (1.643–4.568)	0.022	1.523 (0.987–3.126)	0.044	1.386 (0.921–1.953)	0.084
FOXM1cyto	0.763 (0.458–1.034)	0.770	−	−	0.637 (0.357–0.998)	0.624	−	−
FOXM1nucl	1.347 (0.778–1.995)	0.035	0.876 (0.580–1.426)	0.359	1.268 (0.795–2.101)	0.038	0.741 (0.411–1.132)	0.608
FOXO3Acyto	0.595 (0.310–0.872)	0.914	−	−	0.624 (0.398–1.013)	0.386	−	−
Ki67	1.298 (0.756–1.741)	0.045	1.021 (0.616–1.442)	0.167	1.545 (0.977–2.978)	0.034	1.247 (0.915–1.868)	0.089
LIVIN	0.692 (0.482–1.145)	0.781	-	-	0.793 (0.586–1.335)	0.375	-	-
LOXcyto	0.781 (0.433–1.325)	0.541	−	−	0.763 (0.464–1.250)	0.944	−	−
LOXnucl	1.529 (1.186–3.243)	0.037	1.453 (1.151–2.247)	0.088	1.067 (0.834–1.996)	0.198	−	−
MSI1	0.769 (0.443–1.156)	0.128	−	−	0.849 (0.515–1.131)	0.210	−	−
NFKBP65cyto	1.530 (1.126–2.898)	0.015	1.312 (0.949–2.356)	0.054	1.253 (0.881–2.440)	0.045	0.623 (0.418–1.037)	0.476
NFKBP65nucl	0.629 (0.318–0.925)	0.498	-	-	0.722 (0.421–1.214)	0.445	-	-
P53	0.884 (0.569–1.217)	0.115	-	-	0.865 (0.510–1.237)	0.315	-	-
P73cyto	0.887 (0.536–1.213)	0.452	-	-	0.689 (0.480–1.121)	0.766	-	-
P130cyto	0.659 (0.347–1.224)	0.420	−	−	1.035 (0.626–1.435)	0.418	−	−
PINCH	0.720 (0.451–1.279)	0.527	−	−	4.153 (1.672–6.783)	0.001	3.879 (1.525–5.963)	0.005
PPARcyto	1.586 (0.926–3.310)	0.037	1.325 (0.915–2.256)	0.067	0.645 (0.416–1.275)	0.982	−	−
PPARstrom	0.854 (0.571–1.401)	0.429	−	−	0.717 (0.426–1.319)	0.972	−	−
PRL	0.530 (0.352–1.011)	0.519	−	−	0.601 (0.421–1.009)	0.449	−	−
RBM3cyto	0.648 (0.379–1.096)	0.433	−	−	1.326 (0.919–2.148)	0.046	0.788 (0.513–1.345)	0.218
RBM3nucl	0.516 (0.405–1.107)	0.734	−	−	0.753 (0.452–1.130)	0.625	−	−
SATB1	0.858 (0.532–1.610)	0.167	−	−	1.561 (1.022–2.962)	0.048	1.453 (0.986–2.207)	0.094
SIRT6cyto	0.653 (0.420–0.933)	0.433	−	−	0.661 (0.392–0.946)	0.714	−	−
SIRT6nucl	0.715 (0.514–1.211)	0.840	−	−	0.803 (0.568–1.315)	0.419	−	−
TAZ	1.335 (0.898–2.312)	0.039	0.737 (0.512–1.210)	0.628	0.783 (0.448–1.213)	0.424	−	−
WRAP53cyto	1.211 (0.865–2.074)	0.049	0.696 (0.347–1.101)	0.645	3.762 (1.564–5.568)	0.003	3.556 (1.512–5.210)	0.007
WRAP53nucl	0.641 (0.368–1.105)	0.698	−	−	1.527 (1.189–2.991)	0.040	1.463 (1.032–2.313)	0.077
WRAP53stromcyto	0.550 (0.312–1.069)	0.526	−	−	0.613 (0.338–1.195)	0.471	−	−
WRAP53stromnucl	0.677 (0.481–1.156)	0.873	−	−	0.862 (0.501–1.357)	0.394	−	−

CI, confidence interval; HR, hazard ratio; RT, radiotherapy.

## Supplementary Materials

The following are available online at https://www.mdpi.com/2075-4426/10/4/168/s1, Figure S1: RT impact on biomarkers in PT analyzed by Pearson chi-square, Table S1: Clinicopathological characteristics of patients in RT group and Non-RT group.
