# Supplementary material for: Predictive Role of Biopsy Based Biomarkers for Radiotherapy Treatment in Rectal Cancer
Source: J Pers Med. 2020 Oct 13;10(4):168. doi: 10.3390/jpm10040168 (PMC7712120; doi:10.3390/jpm10040168)
Supplement: Supplementary file 1 [file jpm-10-00168-s001.zip › supplementary/S1 Table.docx]

**S1 Table** Clinicopathological characteristics of patients in RT group and Non-RT group

| Characteristics | RT group (%) | Non-RT group (%) | *P* value |
| --- | --- | --- | --- |
| Gender | | | |
| Male | 75 (59.1) | 55 (59.8) | 0.914 |
| Female | 52 (40.9) | 37 (40.2) |  |
| Age (years) | | | |
| ≤67 | 56 (44.1) | 40 (43.5) | 0.928 |
| >67 | 71 (55.9) | 52 (56.5) |  |
| TNM stage | | | |
| I | 15 (11.8) | 11 (12.0) | 0.829 |
| II | 45 (35.4) | 36 (39.1) |  |
| III | 58 (45.7) | 41 (44.6) |  |
| IV | 9 (7.1) | 4 (4.3) |  |
| Differentiation | | | |
| Well | 17 (13.4) | 12 (13.0) | 0.712 |
| Moderately | 78 (61.4) | 61 (66.3) |  |
| Poorly | 32 (25.2) | 19 (20.7) |  |
| Number of tumors | | | |
| Single | 120 (94.5) | 85 (92.4) | 0.531 |
| Multiple | 7 (5.5) | 7 (7.6) |  |
| Surgical type | | | |
| Anterior resection | 87(68.5) | 60 (65.2) | 0.803 |
| Abdominoperineal resection | 35 (27.6) | 29 (31.5) |  |
| Hartmann’s procedure | 5 (3.9) | 3 (3.3) |  |
| Resection margin | | | |
| Tumor free | 117 (92.1) | 83 (90.2) | 0.620 |
| Tumor | 10 (7.9) | 9 (9.8) |  |
| To anal verge (cm) |  |  |  |
| Mean | 8.1 | 8.2 | 1.000 |

RT, radiotherapy
