# Supplementary material for: Predictive Role of Biopsy Based Biomarkers for Radiotherapy Treatment in Rectal Cancer
Source: J Pers Med. 2020 Oct 13;10(4):168. doi: 10.3390/jpm10040168 (PMC7712120; doi:10.3390/jpm10040168)
Supplement: Supplementary file 1 [file jpm-10-00168-s001.zip › supplementary/S2 Table .docx]

**S2 Table.** Comparing the expression of biomarkers between BS and PT in Non-RT group or RT group

| Marker | BS | | PT | | *P* value |
| --- | --- | --- | --- | --- | --- |
|  | *N* | Mean Rank | *N* | Mean Rank |  |
| AEG1-NonRT | 58 | 43.25 | 46 | 49.75 | 0.220 |
| AEG1-RT | 42 | 39.52 | 37 | 36.48 | 0.316 |
| CD163-NonRT | 58 | 41.98 | 43 | 48.23 | 0.054 |
| CD163-RT | 43 | 37.29 | 39 | 35.64 | 0.642 |
| COX2-NonRT | 44 | 38.12 | 37 | 37.89 | 0.547 |
| COX2-RT | 41 | 36.81 | 35 | 37.25 | 0.376 |
| FOXM1cyto-NonRT | 56 | 40.17 | 44 | 51.07 | **0.031** |
| FOXM1cyto-RT | 42 | 32.56 | 35 | 34.34 | 0.202 |
| FOXM1nucl-NonRT | 54 | 51.32 | 45 | 39.68 | 0.003 |
| FOXM1nucl-RT | 41 | 35.14 | 44 | 32.77 | 0.234 |
| FOXO3Acyto-NonRT | 54 | 37.78 | 46 | 52.88 | **<0.001** |
| FOXO3Acyto-RT | 43 | 35.14 | 36 | 33.04 | 0.162 |
| Ki67-NonRT | 45 | 40.13 | 46 | 41.26 | 0.210 |
| Ki67-RT | 28 | 29.12 | 26 | 27.34 | 0.137 |
| LIVIN-NonRT | 59 | 37.78 | 46 | 52.88 | **0.027** |
| LIVIN-RT | 42 | 30.21 | 30 | 29.80 | 0.923 |
| LOXcyto-NonRT | 57 | 53.90 | 46 | 39.10 | 0.002 |
| LOXcyto-RT | 40 | 36.16 | 32 | 24.84 | 0.021 |
| LOXnucl-NonRT | 57 | 57.61 | 48 | 37.81 | <0.001 |
| LOXnucl-RT | 42 | 37.50 | 40 | 24.50 | 0.002 |
| MSI1-NonRT | 57 | 52.13 | 79 | 80.31 | **<0.001** |
| MSI1-RT | 43 | 47.62 | 68 | 61.83 | **0.011** |
| NFKBP65cyto-NonRT | 56 | 34.12 | 46 | 58.88 | **<0.001** |
| NFKBP65cyto-RT | 43 | 20.82 | 35 | 30.18 | **0.012** |
| NFKBP65nucl-NonRT | 56 | 58.50 | 46 | 34.50 | <0.001 |
| NFKBP65nucl-RT | 43 | 51.50 | 35 | 19.50 | <0.001 |
| P53-NonRT | 45 | 48.31 | 47 | 49.68 | 0.326 |
| P53-RT | 29 | 30.14 | 28 | 28.99 | 0.278 |
| P73cyto-NonRT | 58 | 28.00 | 43 | 40.26 | **0.001** |
| P73cyto-RT | 48 | 27.50 | 42 | 47.11 | **<0.001** |
| P130cyto-NonRT | 52 | 50.45 | 50 | 50.55 | 1.000 |
| P130cyto-RT | 42 | 41.45 | 41 | 41.55 | 1.000 |
| PINCH-NonRT | 56 | 48.06 | 46 | 45.88 | 0.533 |
| PINCH-RT | 48 | 41.34 | 43 | 44.59 | 0.352 |
| PPARcyto-NonRT | 54 | 39.02 | 44 | 47.77 | 0.087 |
| PPARcyto-RT | 42 | 39.80 | 36 | 35.02 | 0.237 |
| PPARstrom-NonRT | 54 | 38.71 | 44 | 48.07 | 0.068 |
| PPARstrom-RT | 43 | 35.88 | 36 | 40.37 | 0.278 |
| PRL-NonRT | 56 | 44.57 | 47 | 49.43 | 0.097 |
| PRL-RT | 40 | 39.89 | 37 | 39.11 | 0.796 |
| RBM3cyto-NonRT | 58 | 46.93 | 47 | 49.10 | 0.665 |
| RBM3cyto-RT | 41 | 42.06 | 38 | 34.09 | 0.015 |
| RBM3nucl-NonRT | 57 | 41.92 | 43 | 44.06 | 0.674 |
| RBM3nucl-RT | 42 | 43.98 | 38 | 32.23 | 0.004 |
| SATB1-NonRT | 51 | 37.50 | 41 | 45.50 | 0.077 |
| SATB1-RT | 40 | 39.50 | 41 | 45.20 | 0.091 |
| SIRT6cyto-NonRT | 55 | 54.16 | 46 | 38.84 | 0.003 |
| SIRT6cyto-RT | 44 | 39.38 | 35 | 31.62 | 0.043 |
| SIRT6nucl-NonRT | 53 | 58.08 | 47 | 36.16 | <0.001 |
| SIRT6nucl-RT | 45 | 36.04 | 36 | 17.62 | <0.001 |
| TAZ-NonRT | 57 | 35.87 | 43 | 51.13 | **0.002** |
| TAZ-RT | 44 | 27.98 | 30 | 33.02 | 0.230 |
| WRAP53cyto-NonRT | 54 | 43.69 | 43 | 43.31 | 0.936 |
| WRAP53cyto-RT | 44 | 38.29 | 36 | 34.71 | 0.359 |
| WRAP53nucl-NonRT | 55 | 42.69 | 44 | 45.28 | 0.613 |
| WRAP53nucl-RT | 44 | 36.85 | 35 | 36.12 | 0.856 |
| WRAP53stromcyto-NonRT | 56 | 48.23 | 51 | 50.01 | 0.483 |
| WRAP53stromcyto-RT | 43 | 35.78 | 34 | 35.29 | 0.572 |
| WRAP53stromnucl-NonRT | 53 | 49.47 | 51 | 47.88 | 0.365 |
| WRAP53stromnucl-RT | 45 | 37.98 | 35 | 36.52 | 0.296 |

BS, biopsy samples; N, number of cases; PT, primary tumors; RT, radiotherapy
