# Supplementary material for: Predictive Role of Biopsy Based Biomarkers for Radiotherapy Treatment in Rectal Cancer
Source: J Pers Med. 2020 Oct 13;10(4):168. doi: 10.3390/jpm10040168 (PMC7712120; doi:10.3390/jpm10040168)
Supplement: Supplementary file 1 [file jpm-10-00168-s001.zip › supplementary/S3 Table .docx]

**S3 Table.** Comparing expression of biomarkers between BS and LNM in Non-RT group or RT group

| Marker | BS | | LNM | | *P* value |
| --- | --- | --- | --- | --- | --- |
|  | *N* | Mean Rank | *N* | Mean Rank |  |
| AEG1-NonRT | 58 | 39.85 | 27 | 49.76 | 0.070 |
| AEG1-RT | 42 | 30.63 | 19 | 31.82 | 0.800 |
| CD163-NonRT | 58 | 42.16 | 31 | 50.31 | **0.017** |
| CD163-RT | 43 | 31.21 | 20 | 33.70 | 0.448 |
| COX2-NonRT | 44 | 27.84 | 17 | 39.18 | **0.017** |
| COX2-RT | 41 | 24.74 | 12 | 34.71 | **0.025** |
| FOXM1cyto-NonRT | 56 | 42.16 | 30 | 46.00 | 0.466 |
| FOXM1cyto-RT | 42 | 31.63 | 19 | 29.61 | 0.643 |
| FOXM1nucl-NonRT | 54 | 45.99 | 30 | 36.22 | 0.020 |
| FOXM1nucl-RT | 41 | 31.40 | 18 | 26.81 | 0.269 |
| FOXO3Acyto-NonRT | 54 | 37.37 | 29 | 50.62 | **0.005** |
| FOXO3Acyto-RT | 43 | 31.41 | 22 | 36.11 | 0.284 |
| Ki67-NonRT | 45 | 37.62 | 28 | 34.78 | 0.169 |
| Ki67-RT | 28 | 26.43 | 25 | 24.51 | 0.223 |
| LIVIN-NonRT | 59 | 45.05 | 29 | 43.38 | 0.758 |
| LIVIN-RT | 42 | 32.75 | 21 | 30.50 | 0.626 |
| LOXcyto-NonRT | 57 | 36.44 | 31 | 59.32 | **<0.001** |
| LOXcyto-RT | 40 | 24.75 | 16 | 37.88 | **0.002** |
| LOXnucl-NonRT | 57 | 41.78 | 28 | 45.48 | 0.478 |
| LOXnucl-RT | 42 | 31.26 | 19 | 30.42 | 0.851 |
| MSI1-NonRT | 57 | 42.82 | 29 | 44.84 | 0.699 |
| MSI1-RT | 43 | 32.20 | 21 | 33.12 | 0.839 |
| NFKBP65cyto-NonRT | 56 | 35.88 | 28 | 55.73 | **<0.001** |
| NFKBP65cyto-RT | 43 | 29.59 | 19 | 35.82 | 0.182 |
| NFKBP65nucl-NonRT | 56 | 48.93 | 29 | 31.55 | <0.001 |
| NFKBP65nucl-RT | 43 | 36.12 | 19 | 21.05 | <0.001 |
| P53-NonRT | 45 | 35.20 | 25 | 33.59 | 0.514 |
| P53-RT | 29 | 25.63 | 24 | 24.48 | 0.387 |
| P73cyto-NonRT | 58 | 32.99 | 18 | 56.25 | **<0.001** |
| P73cyto-RT | 48 | 26.62 | 13 | 47.15 | **<0.001** |
| P130cyto-NonRT | 52 | 36.23 | 18 | 33.39 | 0.355 |
| P130cyto-RT | 42 | 29.19 | 15 | 28.47 | 0.839 |
| PINCH-NonRT | 56 | 35.57 | 19 | 45.16 | 0.062 |
| PINCH-RT | 48 | 29.45 | 15 | 40.17 | **0.029** |
| PPARcyto-NonRT | 54 | 39.96 | 28 | 44.46 | 0.382 |
| PPARcyto-RT | 42 | 32.07 | 17 | 24.88 | 0.121 |
| PPARstrom-NonRT | 54 | 37.46 | 27 | 48.07 | **0.043** |
| PPARstrom-RT | 43 | 28.65 | 19 | 37.95 | 0.051 |
| PRL-NonRT | 56 | 33.00 | 17 | 50.18 | **0.002** |
| PRL-RT | 40 | 28.18 | 13 | 23.38 | 0.313 |
| RBM3cyto-NonRT | 58 | 46.01 | 28 | 38.30 | 0.114 |
| RBM3cyto-RT | 41 | 34.95 | 21 | 24.76 | 0.009 |
| RBM3nucl-NonRT | 57 | 43.59 | 26 | 38.52 | 0.352 |
| RBM3nucl-RT | 42 | 36.58 | 23 | 26.46 | 0.030 |
| SATB1-NonRT | 51 | 44.88 | 27 | 29.33 | 0.001 |
| SATB1-RT | 40 | 29.45 | 14 | 21.93 | 0.047 |
| SIRT6cyto-NonRT | 55 | 48.92 | 29 | 30.33 | <0.001 |
| SIRT6cyto-RT | 44 | 37.24 | 20 | 22.08 | 0.001 |
| SIRT6nucl-NonRT | 53 | 47.01 | 27 | 27.72 | <0.001 |
| SIRT6nucl-RT | 45 | 38.50 | 20 | 20.62 | <0.001 |
| TAZ-NonRT | 57 | 41.37 | 27 | 44.89 | 0.506 |
| TAZ-RT | 44 | 31.69 | 21 | 35.74 | 0.374 |
| WRAP53cyto-NonRT | 54 | 45.20 | 28 | 34.36 | 0.035 |
| WRAP53cyto-RT | 44 | 33.43 | 20 | 30.45 | 0.528 |
| WRAP53nucl-NonRT | 55 | 43.30 | 27 | 37.83 | 0.304 |
| WRAP53nucl-RT | 44 | 33.62 | 21 | 31.69 | 0.685 |
| WRAP53stromcyto-NonRT | 56 | 42.59 | 28 | 42.32 | 0.959 |
| WRAP53stromcyto-RT | 43 | 33.76 | 21 | 29.93 | 0.379 |
| WRAP53stromnucl-NonRT | 53 | 41.30 | 27 | 38.93 | 0.486 |
| WRAP53stromnucl-RT | 45 | 35.48 | 22 | 30.98 | 0.134 |

BS, biopsy samples; LNM, metastatic lymph node; N: number of cases; RT, radiotherapy
