# Supplementary material for: Predictive Role of Biopsy Based Biomarkers for Radiotherapy Treatment in Rectal Cancer
Source: J Pers Med. 2020 Oct 13;10(4):168. doi: 10.3390/jpm10040168 (PMC7712120; doi:10.3390/jpm10040168)
Supplement: Supplementary file 1 [file jpm-10-00168-s001.zip › supplementary/S4 Table.docx]

**S4 Table.** Univariate and multivariate Cox proportional hazard models for local/distal recurrence of different biomarkers in BS

|  | RT group | | | | |  | Non-RT group | | | | |
| --- | --- | --- | --- | --- | --- | --- | --- | --- | --- | --- | --- |
|  | Univariate analysis | |  | Multivariate analysis | |  | Univariate analysis | |  | Multivariate analysis | |
|  | HR (95%CI) | *P* |  | HR (95%CI) | *P* |  | HR (95%CI) | *P* |  | HR (95%CI) | *P* |
| AEG1 | 1.503(1.169-2.647) | **0.031** |  | 1.425(1.104-2.187) | **0.071** |  | 0.869(0.510-1.462) | 0.524 |  | - | - |
| CD163 | 0.687(0.389-1.134) | 0.241 |  | - | - |  | 0.611(0.357-1.198) | 0.516 |  | - | - |
| COX2 | 3.226(1.615-4.327) | **<0.001** |  | 2.747(1.733-3.562) | **<0.001** |  | 1.526(1.187-2.235) | **0.032** |  | 1.497(1.091-1.832) | **0.075** |
| FOXM1cyto | 0.738(0.424-1.233) | 0.660 |  | - | - |  | 0.710(0.408-1.215) | 0.545 |  | - | - |
| FOXM1nucl | 1.368(0.887-2.134) | **0.041** |  | 0.796(0.527-1.530) | 0.413 |  | 1.228(0.810-2.054) | **0.047** |  | 0.776(0.439-1.353) | 0.628 |
| FOXO3Acyto | 0.654(0.413-0.995) | 0.827 |  | - | - |  | 0.680(0.376-1.149) | 0.421 |  | - | - |
| Ki67 | 0.828(0.439-1.107) | 0.142 |  | - | - |  | 0.710(0.443-1.028) | 0.357 |  | - | - |
| LIVIN | 0.637(0.379-1.236) | 0.653 |  | - | - |  | 0.712(0.504-1.498) | 0.268 |  | - | - |
| LOXcyto | 0.725(0.416-1.419) | 0.462 |  | - | - |  | 0.757(0.428-1.382) | 0.815 |  | - | - |
| LOXnucl | 1.626(1.211-2.673) | **0.023** |  | 1.448(1.112-2.239) | **0.080** |  | 1.067(0.713-1.701) | 0.264 |  | - | - |
| MSI1 | 0.871(0.511-1.210) | 0.320 |  | - | **-** |  | 0.826(0.504-1.106) | 0.249 |  | - | - |
| NFKBP65cyto | 2.104(1.558-3.762) | **<0.001** |  | 1.922(1.507-3.338) | **<0.001** |  | 1.614(1.217-2.965) | **0.044** |  | 1.301(1.002-2.170) | **0.077** |
| NFKBP65nucl | 0.684(0.369-1.107) | 0.375 |  | - | - |  | 0.749(0.458-1.471) | 0.526 |  | - | - |
| P53 | 0.910(0.564-1.313) | 0.164 |  | - | - |  | 0.886(0.493-1.218) | 0.417 |  | - | - |
| P73cyto | 0.836(0.515-1.403) | 0.531 |  | - | - |  | 0.642(0.422-1.237) | 0.652 |  | - | - |
| P130cyto | 1.521(1.203-2.121) | **0.045** |  | 1.445(1.271-1.842) | **0.066** |  | 1.916(1.461-3.034) | **<0.001** |  | 1.897(1.679-2.516) | **<0.001** |
| PINCH | 0.973(0.617-1.436) | 0.125 |  | - | - |  | 1.515(1.028-2.237) | **0.010** |  | 1.341(0.989-1.734) | **0.063** |
| PPARcyto | 2.875(1.965-4.118) | **<0.001** |  | 2.910(2.091-4.830) | **<0.001** |  | 1.696(1.307-2.515) | **0.042** |  | 1.510(1.284-2.031) | **0.081** |
| PPARstrom | 0.830(0.556-1.537) | 0.538 |  | - | - |  | 0.746(0.418-1.540) | 0.723 |  | - | - |
| PRL | 0.627(0.334-1.049) | 0.426 |  | - | - |  | 0.667(0.416-1.148) | 0.524 |  | - | - |
| RBM3cyto | 0.679(0.362-1.185) | 0.527 |  | - | - |  | 0.553(0.364-1.285) | 0.659 |  | - | - |
| RBM3nucl | 0.735(0.411-1.380) | 0.667 |  | - | - |  | 0.722(0.408-1.617) | 0.586 |  | - | - |
| SATB1 | 0.795(0.466-1.337) | 0.218 |  | - | - |  | 1.580(1.068-2.479) | **0.030** |  | 1.263(0.634-1.798) | 0.291 |
| SIRT6cyto | 0.687(0.415-0.995) | 0.897 |  | - | - |  | 0.624(0.367-1.182) | 0.682 |  | - | - |
| SIRT6nucl | 1.524(1.119-2.831) | **0.041** |  | 1.210(0.782-1.568) | 0.159 |  | 0.620(0.387-1.407) | 0.510 |  | - | - |
| TAZ | 0.755(0.501-1.258) | 0.511 |  | - | - |  | 0.741(0.429-1.353) | 0.537 |  | - | - |
| WRAP53cyto | 1.267(0.734-1.976) | **0.042** |  | 0.584(0.326-1.378) | 0.423 |  | 1.582(1.214-3.428) | **0.020** |  | 1.510(1.203-2.901) | **0.056** |
| WRAP53nucl | 0.607(0.416-1.425) | 0.518 |  | - | - |  | 1.478(1.154-2.997) | **0.046** |  | 1.312(0.627-1.835) | 0.172 |
| WRAP53stromcyto | 0.634(0.359-1.247) | 0.433 |  | - | - |  | 0.724(0.399-1.404) | 0.525 |  | - | - |
| WRAP53stromnucl | 0.782(0.465-1.436) | 0.656 |  | - | - |  | 0.818(0.467-1.519) | 0.793 |  | - | - |

BS, biopsy samples; CI, confidence interval; HR, hazard ratio; RT, radiotherapy
