# Supplementary figures and images for: Predictive Role of Biopsy Based Biomarkers for Radiotherapy Treatment in Rectal Cancer
Source: J Pers Med. 2020 Oct 13;10(4):168. doi: 10.3390/jpm10040168 (PMC7712120; doi:10.3390/jpm10040168)

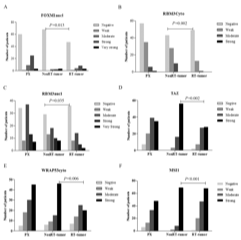

Supplement: Supplementary file 1 [file jpm-10-00168-s001.zip › supplementary/S1 Fig..tiff]

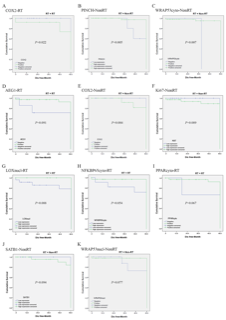

Supplement: Supplementary file 1 [file jpm-10-00168-s001.zip › supplementary/S2 Fig..tiff]

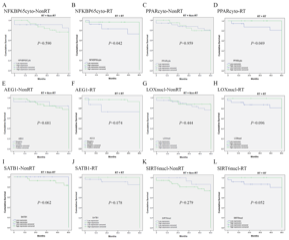

Supplement: Supplementary file 1 [file jpm-10-00168-s001.zip › supplementary/S3 Fig..tiff]

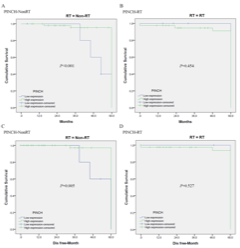

Supplement: Supplementary file 1 [file jpm-10-00168-s001.zip › supplementary/S4 Fig..tiff]
